# Improving the identification of mody mutations by using mlpa technique in the molecular diagnostics routine

**DOI:** 10.1186/1758-5996-7-S1-A246

**Published:** 2015-11-11

**Authors:** Renata Pires Dotto, Andreia Latanza Gomes Mathez, Luciana Ferreira Franco, João Roberto de Sá, Leticia Schwerz Weinert, Sandra Pinho Silveiro, Fernando de Mello Almada Giuffrida, Magnus Regios Dias da Silva, André Fernandes Reis

**Affiliations:** 1 UNIFESP, São Paulo, Brazil

## Background

Maturity-onset diabetes of the young (MODY) represents about 3-5% of cases of diabetes mellitus (DM). Searching for mutations can be performed either by Sanger sequencing or Multiplex Ligation-dependent Probe Amplification (MLPA) technique. MLPA is a powerful molecular tool that identifies large genetic rearrangements such as deletions and insertions, even though these kinds of mutations seem to be rare in the majority of MODY subtypes.

## Objective

To assess the role of the MLPA technique in the genetic screening of GCK-MODY (MODY2), HNF1A-MODY (MODY3) and HNF1B-MODY (MODY5) in cases negative for point mutation using Sanger sequencing.

## Materials and methods

Thirty-one clinically suspected MODY cases according to the guideline criteria that were investigated using Sanger method and were negative for GCK, HNF1A, and HNF1B point mutation, were tested using MLPA. We applied Coffalyser® software for graphical and statistical analysis.

## Results/discussion

Among 12 cases investigated for MODY5, we identify a heterozygous whole deletion of the HNF1B gene in one patient. This patient had a typical phenotype of HNF1B-MODY with familial DM and urogenital tract abnormalities, including renal cysts. He was initially negative for MODY mutation by Sanger sequencing. Although partial gene deletions or duplications account for less than 10% of all disease-causing mutations in hereditary conditions, these mutations can be responsible for up to 40% in HNF1B-MODY. Gross genomic rearrangements are rare in most common forms of MODY such as GCK-MODY and HNF1A-MODY.

## Conclusion

MLPA should be added in molecular diagnostics routine for HNF1B-MODY, in which this technique improves the identification of large genetic rearrangements frequently observed in this MODY subtype.

